# Development of whole-cell catalyst system for sulfide biotreatment based on the engineered haloalkaliphilic bacterium

**DOI:** 10.1186/s13568-021-01302-9

**Published:** 2021-10-24

**Authors:** Manqi Zhang, Qiong Xue, Shengjie Zhang, Heng Zhou, Tong Xu, Jian Zhou, Yanning Zheng, Ming Li, Sumit Kumar, Dahe Zhao, Hua Xiang

**Affiliations:** 1 State Key Laboratory of Microbial Resources, Institute of Microbiology, Chinese Academy of Sciences, 100101 Beijing, China; 2 University of Chinese Academy of Sciences, 100049 Beijing, China; 3 Enzyme and Microbial Biochemistry Lab, Department of Chemistry, Indian Institute of Technology, Delhi, India

**Keywords:** Hydrogen sulfide treatment, Sulfide: quinone oxidoreductase, Haloalkaliphilic heterotrophic bacterium, Genetic modification, Whole-cell catalysis, Process optimization

## Abstract

Microorganisms play an essential role in sulfide removal. Alkaline absorption solution facilitates the sulfide’s dissolution and oxidative degradation, so haloalkaliphile is a prospective source for environmental-friendly and cost-effective biodesulfurization. In this research, 484 sulfide oxidation genes were identified from the metagenomes of the soda-saline lakes and a haloalkaliphilic heterotrophic bacterium *Halomonas salifodinae* IM328 (=CGMCC 22183) was isolated from the same habitat as the host for expression of a representative sequence. The genetic manipulation was successfully achieved through the conjugation transformation method, and sulfide: quinone oxidoreductase gene (*sqr*) was expressed via pBBR1MCS derivative plasmid. Furthermore, a whole-cell catalyst system was developed by using the engineered strain that exhibited a higher rate of sulfide oxidation under the optimal alkaline pH of 9.0. The whole-cell catalyst could be recycled six times to maintain the sulfide oxidation rates from 41.451 to 80.216 µmol·min^−1^·g^−1^ dry cell mass. To summarize, a whole-cell catalyst system based on the engineered haloalkaliphilic bacterium is potentiated to be applied in the sulfide treatment at a reduced cost.

**Supplementary Information:**

The online version contains supplementary material available at 10.1186/s13568-021-01302-9.

## Key points

- Employing a haloalkaliphilic heterotroph as a host for sulfide biotreatment under alkaline conditions.
- Improving the desulfurization rate by engineering the bacterium.
- Developing a recyclable whole-cell catalyst system for the desulfurization process.

## Introduction

Hydrogen sulfide (H_2_S) is the major contaminant in the exploration, transmission, and development of natural gas (Abdelnaby et al. 2020). Because of its highly toxic, malodorous, and corrosive properties, it has detrimental effects on human and animal health (Christia-Lotter et al. 2007) as well as environmental safety (Tang et al. 2009; Shen et al. 2015; Monnot et al. 2017). Various physicochemical and biological technologies for natural gas desulfurization have been developed for decades (Muñoz et al. 2015). There are many problems in the physical and chemical methods, for example, the high temperature, high pressure, and secondary pollution in the absorption (Nowicki et al. 2014; Peluso et al. 2019) and chemical oxidation (Rasi et al. 2011). Furthermore, the membrane separation process is associated with the high cost for the membrane material (Kapdi et al. 2005). By contrast, biological approaches such as THIOPAQ^®^ (Sorokin et al. 2008) and Sulfateq™ (Veolia, France) can convert sulfide to elemental sulfur at normal temperature and pressure (Lin et al. 2018; Schwarz et al. 2020; Flores-Cortés et al. 2021). Moreover, they are cost-effective and environment-friendly (Huisman et al. 2006; Hao et al. 2014; Muñoz et al. 2015) because of low energy consumption, less secondary pollution, high H_2_S removal efficiency, chemical catalysts free in addition to sulfur recovery (de Rink et al. 2020). Therefore, the biodesulfurization process has a broad prospect.

Sulfide oxidizing microorganisms play a significant role in biological sulfide removal processes, and they can be classified into chemoautotroph and chemoheterotroph in terms of carbon and energy sources (Tang et al. 2009). At present, chemoautotrophs are relatively well studied for biodesulfurization. This is because of the fact that they use CO_2_ as a carbon source and inorganic sulfur compounds as a source of energy and reducing power (Muyzer et al. 2013). They do not need the addition of organic substances. *Thiobacillus denitrificans* is a model chemoautotroph microorganism for biodesulfurization. It uses nitrate or nitrite as terminal electron acceptors for sulfide oxidation, resulting in autotrophic denitrification (Mahmood et al. 2007; Beller et al. 2013; Lau et al. 2016). This microbe could remove sulfur-containing compounds coupled with denitrification (Deng et al. 2009; Hao et al. 2019). In this process, the pH value is prone to decrease in the culture medium (Broman et al. 2017). Correspondingly, several *Thiobacillus* species are acidophilic. However, acidification does not favor sulfide absorption for microbial consumption (Hughes et al. 2009) and leads to an increase in cost (Leduc and Ferroni 1994; Oprime et al. 2001).

The alkaline condition weakens the acidification of absorbent and maintains the effective absorption of sulfide. Therefore, alkaliphilic microorganisms have great potential in desulfurization. The genus *Thioalkalivibrio* is a characteristic group of haloalkaliphilic and obligate chemolithoautotrophic sulfur-oxidizing bacteria isolated from soda lake (Sorokin et al. 2001; Janssen et al. 2009). The members of this genus are well adapted to hypersaline (up to salt saturation) and highly alkaline (up to pH 10.5) conditions and fix inorganic carbon as a carbon source (Sorokin et al. 2006). It has been established that the *Vitreoscilla* hemoglobin (VHb) level in *Thioalkalivibrio versutus* D301 promoted thiosulfate oxidation (Mu et al. 2017). The production of nanometric sulfur from sulfide was enhanced by 166.7 % in another *T. versutus* D301 mutant in which the conversion of sulfur to sulfate was blocked by deleting the critical *hdrB* gene (Sharshar et al. 2020). Concisely, it displays remarkable advantages for sulfide removal.

Chemoheterotrophic bacteria are also capable of oxidizing sulfide to the higher valence of sulfur compounds during growth on organic compounds under aerobic conditions (Krayzelova et al. 2015). As previously reported, the sulfide oxidation rates of eight heterotrophic bacteria ranged from 0.1 to 50 µmol·min^−1^·g^−1^ dry cell mass, showing that the rates of sulfide removal are comparable to those of chemoautotrophic bacteria (Hou et al. 2018). Therefore, the heterotrophic bacteria capable of rapidly oxidizing sulfide offer an alternative for sulfide biotreatment (Xia et al. 2017; Xin et al. 2020). Sulfide: quinone oxidoreductase (Sqr) and flavocytochrome *c* sulfide dehydrogenase (FCSD) are two different enzymes with sulfide oxidation activity in the periplasmic and cytosolic sides of the membrane. They can oxidize sulfide to sulfane sulfur (Sousa et al. 2018). Sqrs are classified into six types based on the structural analysis (Marcia et al. 2010). Subsequently, persulfide dioxygenase (Pdo) further oxidizes sulfane sulfur to sulfite and is found in *Cupriavidus pinatubonensis* JMP134 (Xin et al. 2020). Some heterotrophic bacteria contained only Sqr, while others contained both Sqr and Pdo, and they were able to oxidize sulfide (Gao et al. 2017; Xia et al. 2017). Recently, *Spiribacter halalkaliphilus*, an abundant haloalkaliphilic species in Chinese soda-saline lakes, was isolated. This heterotrophic microbe adopts multiple adaptive mechanisms, including the sulfide’s oxidation for additional energy (Xue et al. 2021).

Whole-cell biocatalysis has been widely used for efficient biosynthesis with unique advantages. It not only reduces the cost by averting supplementation of expensive co-factors and protein purification process but also helped to stabilize the enzymes under the harsh reaction conditions (Wu and Li 2018). Furthermore, the cells could be used repeatedly. At present, advances in metabolic engineering and synthetic biology have markedly improved the catalytic efficiency using whole-cell biocatalytic processes (Lin and Tao 2017).

In this work, hundreds of potential sulfide oxidation genes were obtained from the previous metagenomic research of soda-saline lakes. At the same time, a haloalkaliphilic heterotrophic bacterium *Halomonas salifodinae* IM328 with the capability of sulfide oxidation was isolated from the same habitat as the gene expression host. The genetic manipulation system of IM328 was established, and the sulfide oxidation rate was improved by expressing the heterological *sqr*. Subsequently, a whole-cell catalyst system was developed, and the process was optimized to treat sulfide contamination.

## Materials and methods

### Phylogenetic analysis of sulfide oxidation genes from metagenomes of soda-saline lakes

Protein sequences hit of Sqr and FCSD and its corresponding gene abundance were retrieved from the non-redundancy protein catalogue of eighteen soda-saline lake metagenomes described previously (Zhao et al. 2020). Sqr proteins were classified based on the phylogenetic relationship with previously reported reference sequences (Marcia et al. 2010; Sousa et al. 2018). Reference sequences of Sqr and FCSD used for evolutionary analysis are summarized in Additional file 1: Table S1. Protein sequences were aligned with CLUSTAL W (Thompson et al. 1994). Evolutionary analyses were conducted in MEGA X (Kumar et al. 2018) and visualized using iTOL (Letunic and Bork 2019). FCSD sequences formed an outgroup in the phylogenetic tree.

### Isolation, culture conditions and physiological features of haloalkaliphilic strain

The turbid water sample collected from a soda-saline lake in Inner Mongolia was diluted and spread on HM-3.6 solid medium plates. Plates were incubated at 28 °C to get separate individual colonies. The components of the HM-3.6 medium were (g/L): sodium chloride 36, potassium chloride 2, magnesium sulfate heptahydrate 1, calcium chloride 0.27, sodium bromide 0.23, sodium bicarbonate 0.06, peptone 5, yeast extract 10, ferric chloride 0.001, pH 8.5 (adjusted with NaOH), and agar 1.5 % (w/v) for solid medium.

The 16 S rRNA gene of the isolated strain was amplified by standard PCR protocols using the forward primer 27 F and reverse primer 1492R (Table 1). The DNA sequence was determined by Sanger sequencing carried out by GENEWIZ Biotech Company (Suzhou, China). The sequences of the 16 S rRNA gene were compared with 16 S rRNA gene sequences available in EzBioCloud Database. Sequences were aligned with CLUSTAL W (Thompson et al. 1994). Phylogenetic trees were constructed according to the Maximum Likelihood within the MEGA X program package (Kumar et al. 2018). The Evolutionary distance was calculated according to the algorithm of the Tamura-Nei model (Tamura and Nei 1993).

**Table 1:** Primers used in this study

Primer name	Sequence	Description
27 F	AGAGTTTGATCCTGGCTCAG	sequencing of the 16S rRNA gene
1492R	GGTTACCTTGTTACGACTT	sequencing of the 16S rRNA gene
sqr-F	CACTGCAGGAGGAAGCTTATGCCCAACGAATCA	*sqr* amplification
sqr-R	TCCCAGCTCAACGCCCTAGATTCGGCCACG	*sqr* amplification
TER-F	TGCTACGTGGCCGAATCTAGGGCGTTGAGCTGGGATTAACCCGGCGAGGCGGAGACCCAACAGAACGGAGCCAGGGAGATGGCGACGCAG	pBBR-ptac-sqr plasmid construction
TER-R	ACTCGATTGACTGGGGGGCTAGCTGCGTTGAGGAGCCAGCCAGCGCCACTGGGGTCAAACCTTGTCTGTTGACCCTCGTGCCC	pBBR-ptac-sqr plasmid construction
CX-F	ACTGCATAATTCGTGTCGCT	Sequencing primer for pBBR-ck
CX-R	AAGAGGAGCAACGCGATCTA	Sequencing primer for pBBR-ck
H1_Hin_TER-F	AGGACACCTGGGGCACCAACGCCTGAAAGCTTGGCGTTGAGCTGGGATTAACCCGGCG	pBBR-ptac-ck construction

*Escherichia coli* and *H*. *salifodinae* IM328 were cultivated in Luria-Bertani (LB) and LB-60 medium. The LB-60 medium contained sodium chloride 60 g/L, yeast extract 5 g/L, and tryptone 10 g/L. The routine culture conditions were 37 °C with 200 rpm shaking. LB-20 medium was used to incubate the mixture of IM328 and S17-1. Its composition was sodium chloride 20 g/L, yeast extract 5 g/L, and tryptone 10 g/L. LB-80 medium was used to screen transformants, and it contained sodium chloride 80 g/L, yeast extract 5 g/L, and tryptone 10 g/L. To maintain the stability of the plasmid during bacterial growth, chloramphenicol (25 mg/L) was added. The optimal pH conditions for growth were determined in LB-60 medium by adding 25 mM MOPS (pH 7.0), HEPES (pH 7.5), Tricine (pH 8.0-8.5), and CHES (pH 9.0). To prepare the solid medium plate, 1.5 % (w/v) agar was added. The strains and plasmids used in this work are listed in Table 2.

**Table 2:** Strains and plasmids used in this study

Strain or plasmid	Description/characteristic	Source/reference
Strain		
* Escherichia coli* DH5α	Cloning strain	TSINGKE (China, Beijing)
* Escherichia coli* S17-1	A vector donor in conjugation, integrates RP4 derivative in chromosome	Simon (1984)
S17_sqr	Derivate of S17-1, containing plasmid pBBR-ptac-sqr	This study
* Halomonas salifodinae* IM328	Wild type, isolated from a soda-saline lake in China	This study
* H. salifodinae* IM328_sqr	Derivate of IM328, containing plasmid pBBR-ptac-sqr	This study
* H. salifodinae* IM328_ck	Derivate of IM328, containing plasmid pBBR-ptac-ck	This study
Plasmid		
pBBR-ptac-rfp	Derivate of plasmid pBBR1MCS, *tac* promoter, expressing *rfp* gene	Kovach et al. (1995), Mu et al. (2017)
pBBR-ptac-sqr	Derivate of plasmid pBBR1MCS, *tac* promoter, expressing *sqr* from *Spiribacter* sp. IM2438	This study
pBBR-ptac-ck	*rfp* deletion from pBBR-ptac-rfp vector	This study

### Field emission scanning electron microscopy

To observe the morphology of the bacteria, field emission scanning electron microscopy (FESEM) was performed. Cells were harvested by centrifugation at 10,000 g for 10 min and then were fixed overnight at 4 °C with glutaraldehyde in 0.2 M phosphate buffer (pH 8.0) containing 6 % NaCl. Further, cells were washed three times in the same buffer. The specimen was dehydrated using a graded series of ethanol (50 %, 70 %, 85 %, 95 %, and 100 %) for 15 min in each soaking. In the end, the specimen was dehydrated in Leica EM CPD300 automated critical point dryer for 75 min, dried with CO_2_, coated with gold in ion sputter E-1045, and observed using a FESEM Hitachi SU8010 (Hitachi, Tokyo, Japan).

### Molecular biology experiments

The gene encoding region of Sqr was amplified from *Spiribacter* sp. IM2438 (GenBank assembly accession: GCA_009676705.1) using primers sqr-F and sqr-R. A part of the terminator T1 was amplified from pBBR-ptac-rfp (Table 2) using primers TER-F and TER-R. The *sqr* gene and partial T1 were linked by fusion PCR using primers sqr-F and TER-R. The fused fragment was inserted into pBBR-ptac-rfp after double digestion with *Hin*d III and *Nhe* I forming pBBR-ptac-sqr plasmid. The pBBR-ptac-ck plasmid was constructed by deleting *rfp* gene from pBBR-ptac-rfp and was used as the negative control. *E. coli* DH5α was used to construct expression plasmids. All primers sequences are listed in Table 1.

The conjugation method was performed for transformation. *E. coli* S17-1 was used as a vector donor for plasmid pBBR1MCS derivatives. *E. coli* S17-1 and *H*. *salifodinae* IM328 were cultivated in LB medium and LB-60 medium respectively, overnight at 37 °C with shaking (200 rpm). Cells were harvested by centrifugation at 6000 g for 10 min at 4 °C then washed twice with LB (for *E. coli* S17-1) and LB-60 (for IM328). The mixture of S17-1 and IM2438 at a 1:1 ratio was subsequently incubated on LB-20 solid medium at 37 °C for 18 h. The exconjugants were then resuspended and plated on a solid LB-80 medium containing 25 mg/L chloramphenicol. After incubation at 37 °C for 4 days, colonies could be observed on the surface of the solid plate. The transformants containing plasmid pBBR-ptac-sqr and pBBR-ptac-ck were identified by colony PCR using primers sqr-F/sqr-R and CX-F/CX-R, respectively. Briefly, the cells of different strains were added to the PCR reactions as a template source.

Sodium dodecyl sulfate polyacrylamide gel electrophoresis (SDS-PAGE) of total proteins was performed using 12 % separating gel under denaturing conditions. The cells were suspended in lysis buffer and broken by sonication. The resulting extracts were boiled in the 5 × SDS-PAGE loading buffer (Solarbio, Beijing, China) containing β-mercaptoethanol for 10 min. After cooling and centrifugation, the final supernatant was used for gel loading. The protein bands were analyzed after staining.

### Whole-cell catalysis for sulfide oxidation and reuse

The derivatives of IM328 were grown in LB-60 medium with 25 mg/L chloramphenicol overnight at 37 °C. When the OD_600_ reached 4, the cells were harvested by centrifugation (6000 g, 10 min) and suspended in 25 mM tricine buffer (pH 8) at an OD_600_ of 2. For the heat-killed control, the cell suspension was heated in boiling water for 10 min and then cooled to room temperature. One milliliter of the cell suspension was transferred to a 1.5 mL capped tube. A freshly prepared sodium sulfide solution was added to it at a final concentration of 1000 µM to initiate the reaction. The tube was capped tightly and incubated at 37 °C with shaking at 200 rpm.

Sulfide was analyzed at various time intervals by using a diamine reagent (Fogo and Popowsky 1949), containing *N, N*-Dimethyl-p-phenylenediamine dihydrochloride (C_8_H_14_Cl_2_N_2_, 3.8 g/L, Shanghai Yuanye Bio-Technology Co.) and ferric chloride (FeCl_3_·6H_2_O, 56.6 g/L). After this, the absorbance of the solution was recorded spectrophotometrically at 670 nm. The sulfide concentration in the sample was calculated based on the standard curve.

After the reaction, cells were harvested again, washed with 25 mM tricine buffer solution, and lyophilized for 24 h to weigh the dry cell mass. Sulfide oxidation rate (*q*) was determined by the following equation:

$$q\, = \,{{(C_{0} \, - \,C_{t} )\, \times \,V} \mathord{\left/ {\vphantom {{(C_{0} \, - \,C_{t} )\, \times \,V} {\left( {m\, \times \,T} \right)}}} \right. \kern-\nulldelimiterspace} {\left( {m\, \times \,T} \right)}}$$

where *C*_0_ and *C*_*t*_ were the sulfide concentrations (µM) at the beginning and at the end; *V* was the volume of solution (mL), *m* was the dry cell mass (g), and *T* was the reaction time (min).

The optimal pH conditions for sulfide oxidation were determined in whole-cell catalysis buffer solution by adding 25 mM MOPS (for pH 7.0), Tricine (for pH 8.0), CHES (for pH 9.0), and CAPS (for pH 10.0). Under pH 9.0 condition, cells were reused in 1.5 mL tubes at an OD_600_ of 2. Each reaction cycle was carried out for 40 min. Sulfide was added at final concentration of 1000 µM to initiate the reaction in each cycle. Sulfide levels were determined by using the diamine reagent as described above at 5 min intervals.

## Results

### Mining sulfide oxidation genes from metagenomes of soda-saline lakes

**Fig. 1:**
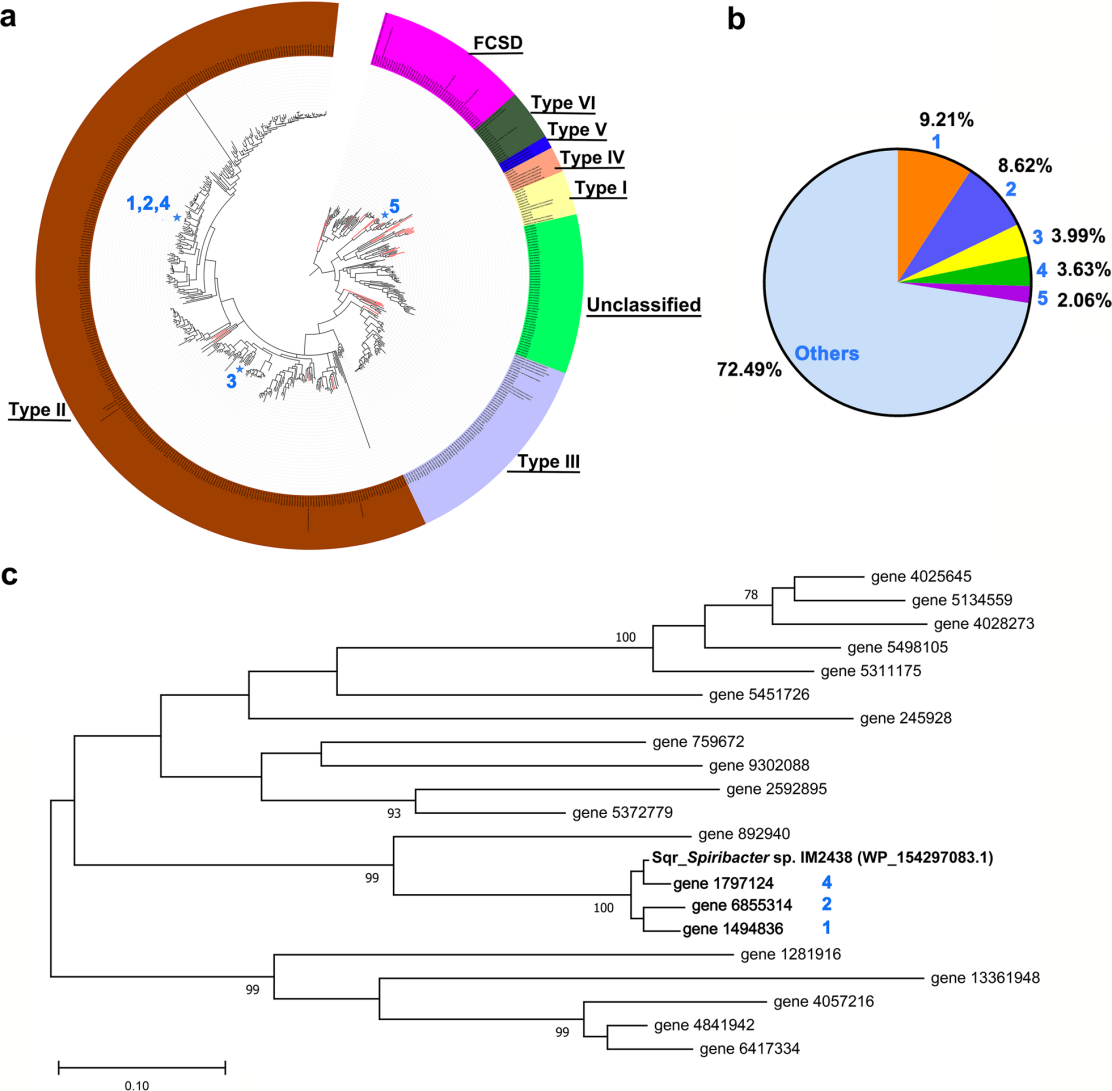
Bioinformatic analysis of sulfide oxidation genes from microbial metagenomes of soda-saline lakes. **a** Phylogenetic analysis of sulfide: quinone oxidoreductase (Sqr) and flavocytochrome c: sulfide dehydrogenase (FCSD). The sequences were obtained from previous research (Zhao et al. 2020). The evolutionary tree was inferred by using the Maximum Likelihood method and JTT matrix-based model (Jones et al. 1992). The tree with the highest log likelihood (-644966.85) is shown. Initial trees for the heuristic search were automatically obtained by applying Neighbor-Join and BioNJ algorithms to a matrix of pairwise distances estimated using the JTT model. Then, the topology with a superior log-likelihood value was selected. There were 986 positions in the final dataset. This analysis involved 484 sulfide oxidases and 33 reference sequences indicated by red branches (All sequence data shown in Additional file 1: Table S1). The outer circle was colored by different Sqr types and FCSD. The positions of the top five Sqrs in abundance were marked with blue five-pointed stars. The blue numbers (1 to 5) were the abundance rankings corresponding to those in Fig. 1b and c. **b** The percentage of the total abundance of the proteins in 18 samples. The abundance data were described in previous work (Zhao et al. 2020) and were shown in Additional file 1: Table S2. **c** Maximum Likelihood phylogenetic tree based on the Sqr of *Spiribacter* sp. IM2438 and the partial type II Sqrs in Fig. 1a (20 protein sequence names shown in red in Additional file 1: Table S1). Bootstrap values (%) were based on 1000 replicates and depicted with more than 70 % bootstrap support. NCBI reference accession numbers are shown in parentheses. Bar, 0.01 substitutions per nucleotide position

To harness the potential of the halophilic microbial and genetic resources, the Sqr and FCSD sequences were retrieved and annotated. A total of 484 amino acid sequences were obtained (details shown in Additional file 1: Table S2). From the further analysis of the evolutionary tree, 440 Sqrs and 44 FCSDs were identified (Fig. 1a). Moreover, Sqr sequences clustered into types I, II, III, IV, VI and an unclassified type. The evolutionary tree indicated that 307 Sqrs belonged to type II, accounting for 63 %. Besides, 10 type I, 57 type III, 3 type IV, and 13 type VI Sqr sequences were annotated, but no type V Sqr sequences were found in this analysis by comparing them to reference protein sequences. It was found that the type II Sqrs were not only the largest in number but also the most abundant genes in soda-saline lake metagenomes. The top four proteins in abundance belonged to type II.

Further, the sequences ranked 1, 2, and 4 were very similar in evolutionary relationships, representing 21.46 % of the total sulfide oxidation genes in the metagenomes of 18 brine and sediment samples (Fig. 1b). They showed a high similarity of 96.15, 95.93, and 98.05 % with the Sqr protein (NCBI reference sequence: WP_154297083.1) of *Spiribacter* sp. IM2438 isolated from the soda-saline lake (Fig. 1c). The abundant Sqr might have better adaptability to saline and alkaline conditions, consequently the *sqr* of *Spiribacter* sp. IM2438 was selected for expression and sulfide treatment.

### A haloalkaliphilic strain for the expression of ***sqr***

**Fig. 2:**
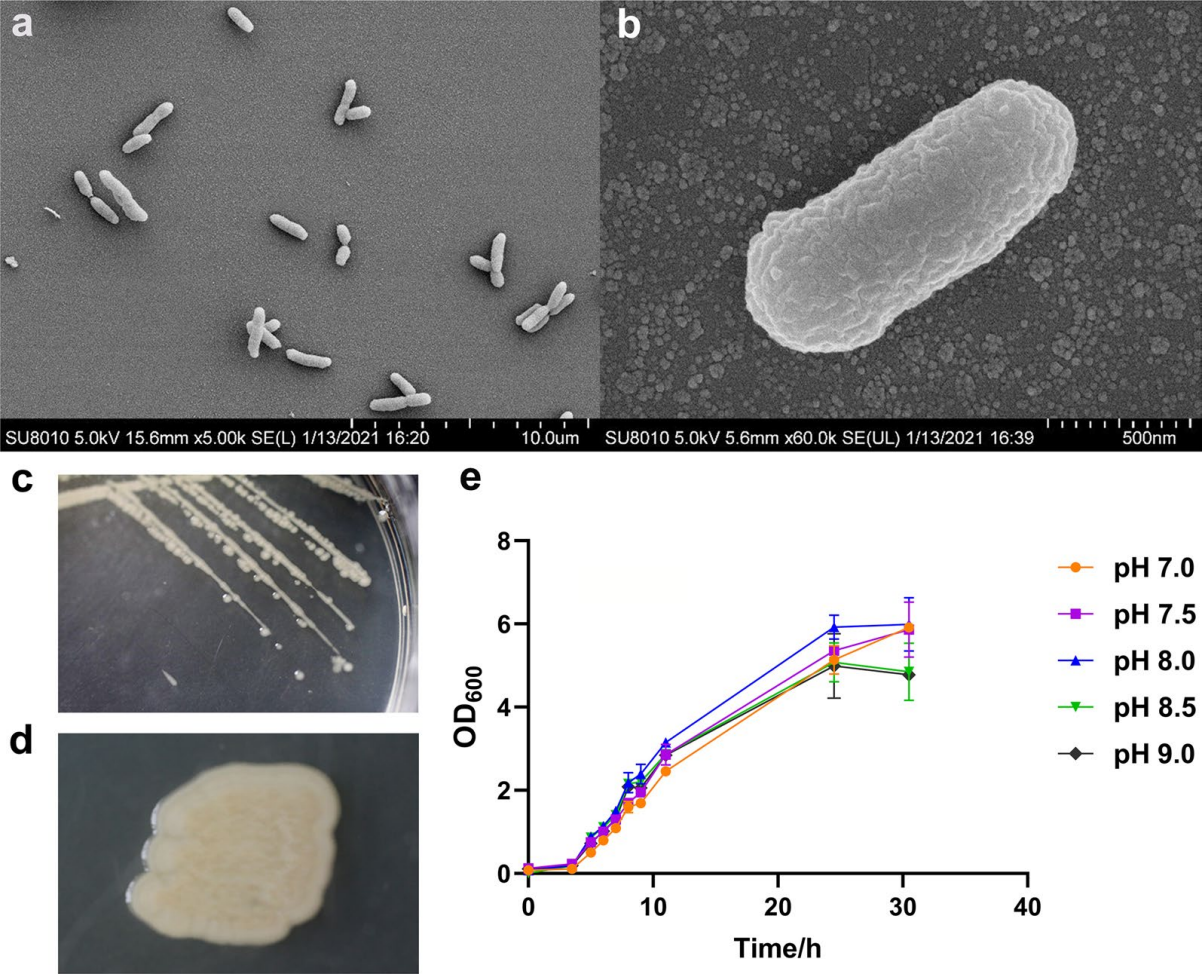
Morphology and physiological characteristics of *Halomonas salifodinae* IM328. Cell morphology of IM328 visualized using a transmission electron microscope (**a, b**), colony morphology on solid medium (**c, d**), and growth curve in media of different pH values (**e**), respectively. IM328 was cultured in LB-60 medium for determining the optimal growth pH by adding 25 mM MOPS (pH 7.0), HEPES (pH 7.5), Tricine (pH 8.0 and 8.5), and CHES (pH 9.0) at 37 °C, 200 rpm. These data were mean values, and standard errors were calculated from three parallel incubations

To express the *sqr* gene, *H. salifodinae* IM328 was used as host. This strain was isolated from the environmental sample collected from soda-saline lake in Inner Mongolia of northern China. Cells were long rods, 0.5-1.0 μm wide and 1.0-2.0 μm long (Fig. 2a and b). Matured colonies on complex agar medium were usually 1-2 mm in diameter, circular, smooth, elevated, and yellow to orange in colour after growth at 37 °C (Fig. 2c and d). The isolate was able to grow in LB-60 medium containing 6 % (w/v) NaCl at pH 7.0–9.0 (optimum at pH 8.0) (Fig. 2e). Considering the alkaliphilic characteristic of IM328, it could be developed as a candidate host for *sqr* overexpression to remove sulfide under alkaline conditions.

**Fig. 3:**
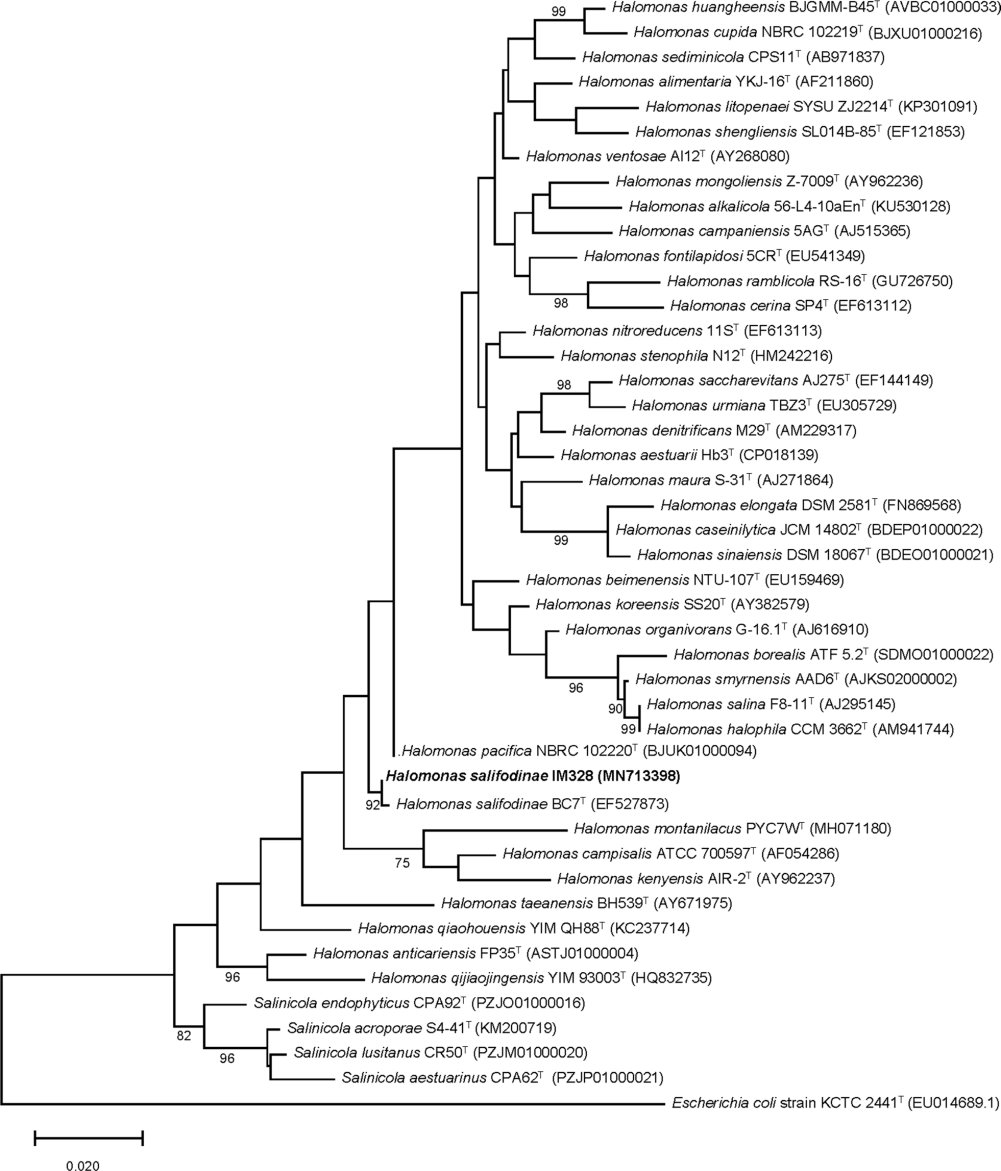
Phylogenetic tree analysis of IM328 strain based on 16 S rRNA gene. This analysis involved 45 nucleotide sequences by the Maximum Likelihood method and Tamura-Nei model (Tamura and Nei 1993). *E*. *coli* strain KCTC 2441^T^ was the outgroup bacterium used for the root of the tree. The sequences and the sequence accession numbers were obtained from GenBank. Evolutionary analyses were conducted in MEGA X (Kumar et al. 2018). Bar, 0.02 substitutions per nucleotide position

To identify the taxonomic classification, the phylogenetic analysis was carried out based on 16 S rRNA gene sequences. The result showed that strain was closely related to the genus *Halomonas* and had the highest sequence similarity to the type strain of *Halomonas salifodinae* BC7^T^ (99.71 %), indicating that this strain belongs to the *Halomonas salifodinae* species (Fig. 3). Phylogenomic tree of concatenated amino acid sequences of conserved proteins supported the taxonomic status (Additional file 2: Fig. S1). Therefore, it was named *H*. *salifodinae* IM328 (=CGMCC 22183). The GenBank accession number for the 16 S ribosomal RNA gene sequence of strain IM328 (1289 bp) is MN713398.

### Construction of the engineered strain for overexpression of ***sqr*** in IM328

**Fig. 4:**
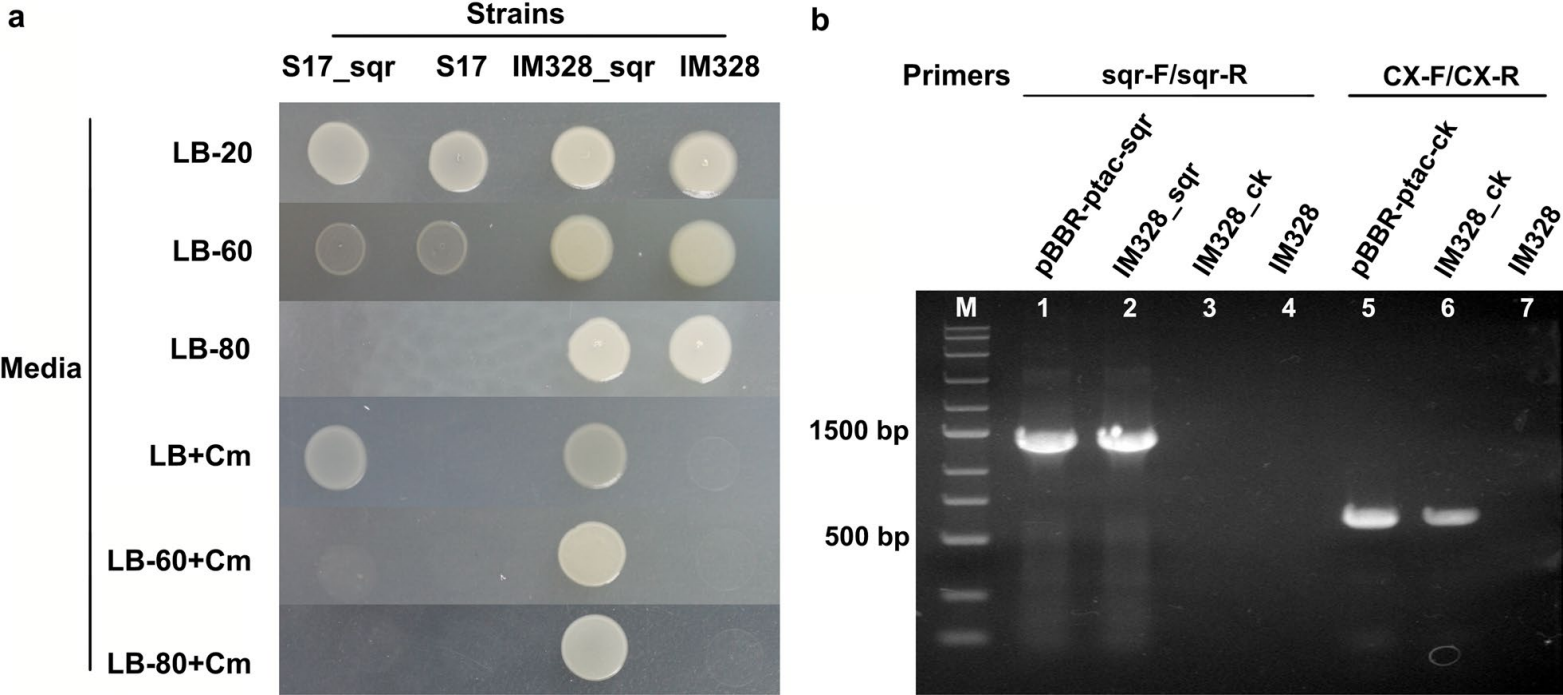
Establishment of IM328 genetic transformation system. **a** The growth of the strains used for transformation on different solid media for screening. Cm: 25 mg/L chloramphenicol was added. **b** Identification of IM328_ck and IM328_sqr strain by colony PCR using primers sqr-F/sqr-R and CX-F/CX-R, respectively

The genetic transformation system was developed using the conjugation transformation method to express the heterologous *sqr* gene in IM328. With *E*. *coli* S17-1 as the donor strain, a plasmid pBBR-ptac-sqr containing *tac* promoter and chloramphenicol resistant gene (Table 2) was transformed into IM328. As IM328 is sensitive to chloramphenicol and *E*. *coli* S17-1 does not grow under 8 % salinity, so we used chloramphenicol containing LB-80 medium with 8 % salinity for selection of the IM328 transformants. In this medium, neither *E*. *coli* S17-1 nor IM328 wild-type grows (Fig. 4a).

The exconjugants were inoculated on the fresh LB-80 with chloramphenicol, and it grew well. To confirm the existence of the pBBR-ptac-sqr plasmid, colony PCR was performed for the different strains using sqr-F/sqr-R primers. A band was observed when the cells of IM328_sqr were added as template source, and the length of PCR product corresponded to the theoretical size of 1362 bp, but there were no bands in the samples of the IM328 and IM328_ck (containing pBBR-ptac-ck as a negative control, Fig. 4b). This result proved that the IM328_sqr strain was successfully constructed. Besides, IM328_ck was identified by colony PCR with primers CX-F/CX-R, and it showed that the product length was also corresponding with the theoretical size of 551 bp (Fig. 4b). Therefore, the genetic transformation system of haloalkaliphilic IM328 was established. The transformants of IM328 were verified to be constructed successfully at the gene level.

**Fig. 5:**
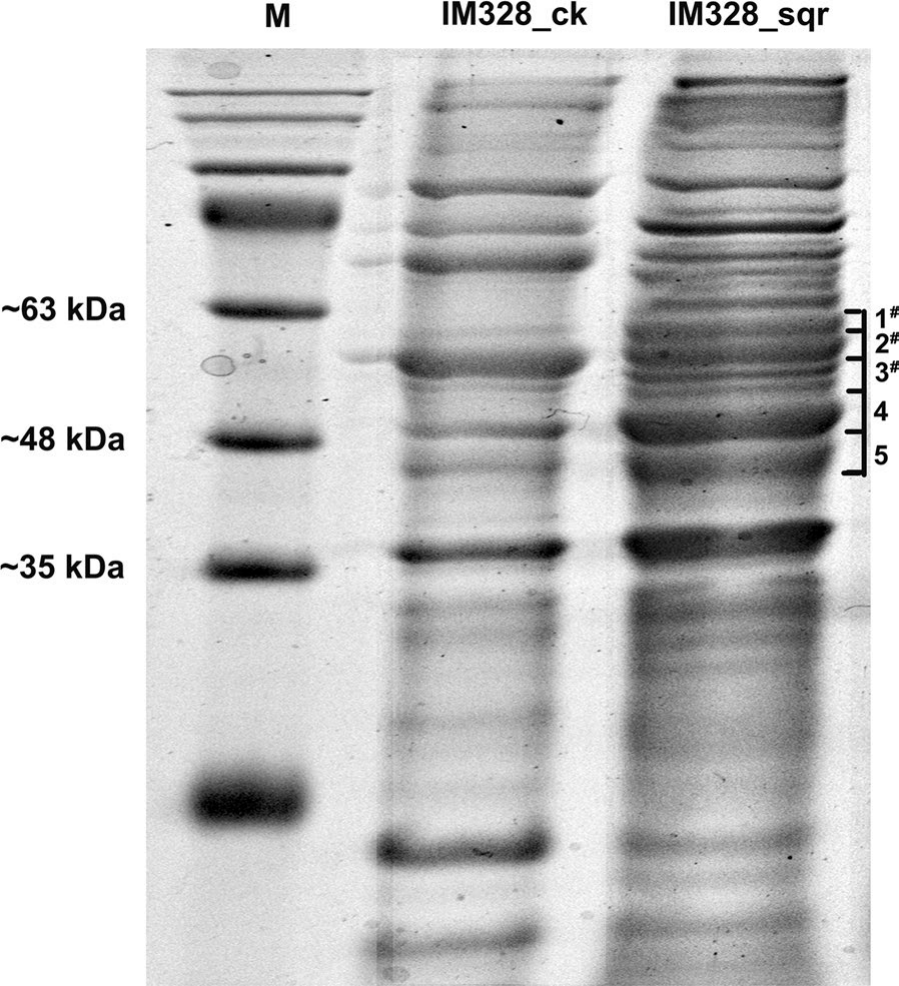
SDS-PAGE of IM328_ck and IM328_sqr. Stacking gel of 5 % polyacrylamide (pH 6.8) and separating gel of 12 % polyacrylamide (pH 8.8) were used, and 20 µL protein extracts were loaded for electrophoresis at the low temperature. The gels containing possible target bands of similar molecular weight were recovered for mass spectrometry, and the samples were numbered 1 to 5, of which samples 1, 2, and 3 (marked by #) contained peptide fragments of interest Sqr. The details are summarized in Additional file 1: Table S3. IM328_ck: IM328_ck strain as control; IM328_sqr: IM328_sqr strain; M: protein marker

The pBBR-ptac-sqr plasmid with tac promoter was introduced into IM328 to express *sqr*. To ensure the expression of *sqr* in the engineered strain, SDS-PAGE was performed. The putative molecular weight of the target protein Sqr is approximately 48.1 kDa. SDS-PAGE showed some differential bands of putative molecular weight in the total proteins of IM328_sqr compared with the control (Fig. 5). Thus the gel bands (numbered 1 to 5) possibly containing the protein of interest were recovered for mass spectrometry (Fig. 5). If the gene was expressed, the peptide fragments could be detected. The mass spectrometry results indicated that there were 33 matched trypsin digestion peptides to the Sqr protein in the No. 2 sample (expectation values of all peptides were less than 0.05), and the protein sequence coverage was 47.4 %. In contrast, there were relatively few matched peptides in samples 1 and 3 (2 and 19 matched peptides, respectively; detailed data is shown in Additional file 1: Table S3). Matching peptides was not observed in the 4 and 5 samples. The result of matching peptides in sample 2 further proved *sqr* gene expression at the protein level.

### Whole-cell catalysis for biodesulfurization

**Fig. 6:**
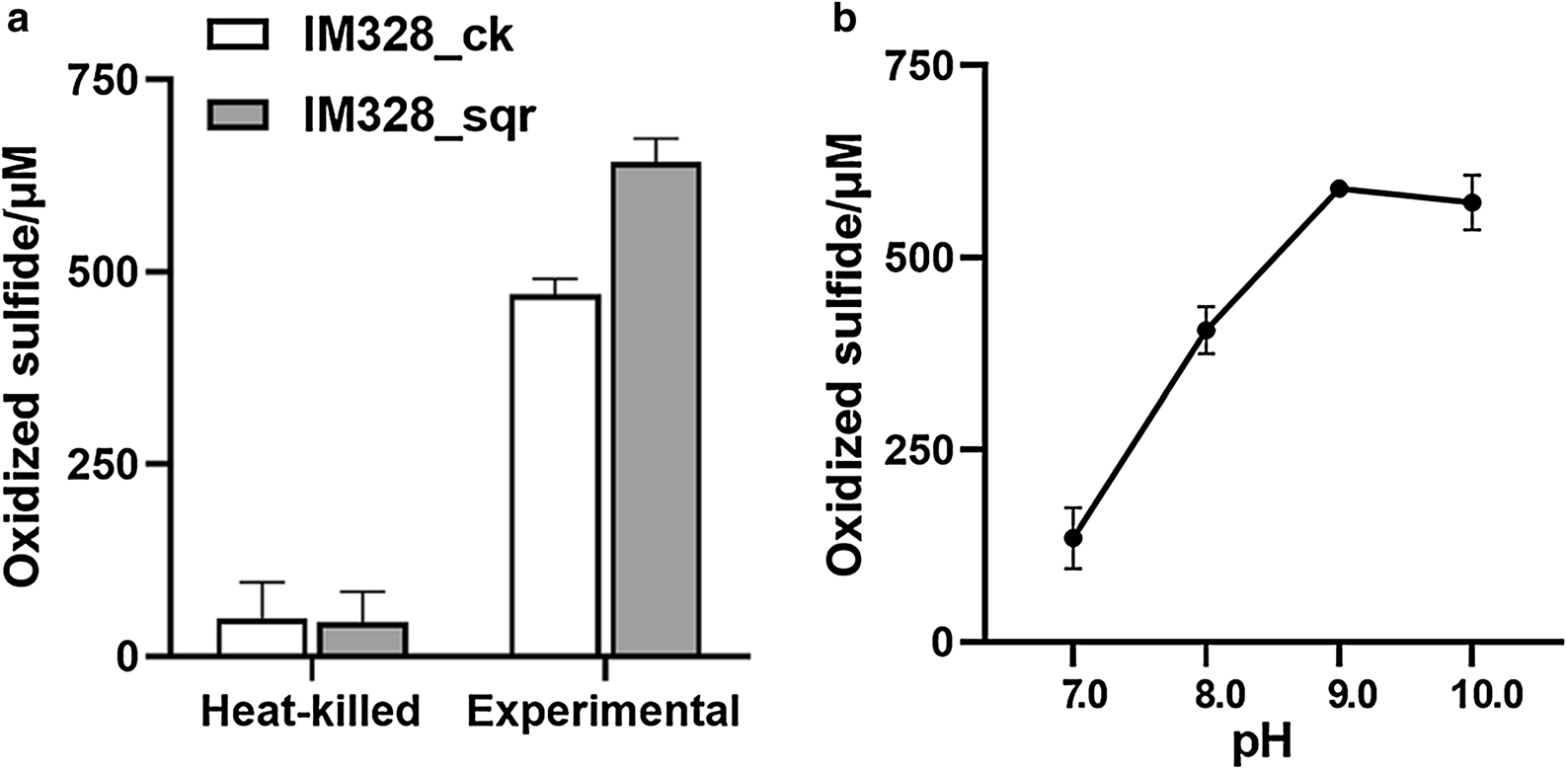
Sulfide oxidation by *H*. *salifodinae* IM328_sqr. The experiments were carried out in the 1.5 mL centrifuge tube. Na_2_S was added to 1000 µM to initiate the reaction. Detection and calculation of the sulfide contents consumed in the reaction system were done after 5 min. **a** Comparison of the desulfurizing activity of IM328_sqr and IM328_ck under aerobic conditions. Sulfide oxidation by live and heated killed cells. Cells were harvested from LB-60, washed and resuspended in 1 mL of 25 mM tricine buffer (pH 8.0) at 37 °C, 200 rpm. The biomass was equivalent for *H*. *salifodinae* IM328_sqr and IM328_ck (OD_600_ of approximate 4). **b** Optimization of the pH value of the whole-cell catalysis of IM328_sqr. Cells were resuspended at OD_600_ of 2 in 25 mM buffer corresponding to different pH values, respectively. Averages (n = 3) with standard deviations (error bar) are shown

To utilize the engineered IM328 strain, a method of whole-cell catalysis was developed. The sulfide oxidation rate was estimated by the reduction of sulfide concentration in the unit time. The standard curve of sulfide concentration is shown in Additional file 2: Fig. S2. After 5 min of reaction, the sulfide oxidation rates of IM328_sqr and IM328_ck were 34.673 and 25.860 µmol·min^−1^·g^−1^ dry cell mass, respectively (Fig. 6a). The sulfide oxidation rate of IM328_sqr was 34.081 % faster than that of the IM328_ck. Sulfide oxidation rate was about 3 µmol·min^−1^·g^−1^ dry cell mass in the heat-killed control experiment with IM328 mutants, and the reduction of sulfide concentration may be attributed to volatilization and abiotic oxidation. The increase of Sqr activity provided the enzymatic evidence that the *sqr* gene was successfully expressed.

IM328_sqr was more efficient for sulfide oxidation. Furthermore, optimization of the pH value for the whole-cell catalytic reaction system using engineered bacteria IM328_sqr was done. It was found that the reaction rate was best at pH 9.0, reaching 77.061 µmol·min^−1^·g^−1^ dry cell mass (Fig. 6b). Therefore, the optimal pH for the whole-cell catalytic reaction is 9.0.

### Stable sulfide oxidation activities of IM328_sqr after recycling

**Fig. 7:**
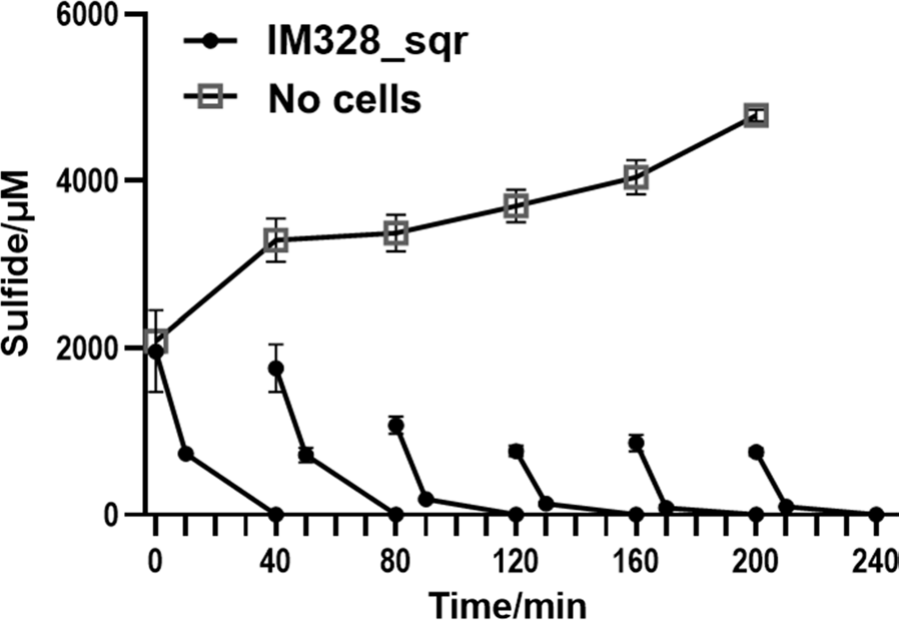
The sulfide oxidation activity of IM328_sqr during six cycles of recycling. The experiments were carried out in a 15 mL centrifuge tube. Cells were resuspended at OD_600_ of 2 in 10 mL of 25 mM CHES buffer (pH 9.0) at 37 °C, 200 rpm. Na_2_S was added to 2000 µM to initiate the reaction in each cycle and was completely oxidized within 40 min. The process of sulfide oxidation was performed for 40 min. The remaining sulfide levels of the reaction system were detected after ten minutes of each cycle. Controls consisted of the same buffer without bacterial cells

It would sharply reduce the cost if the cells of engineered IM328_sqr could be reused, subsequently, the related research was carried out. A total of six cycles were designed, and 2000 µM sodium sulfide was added in each cycle. The result showed that sodium sulfide concentration decreased rapidly in 10 min of reaction until it was completely consumed after 40 min (Fig. 7). The sulfide oxidation rates of the mutant with *sqr* ranged from 41.451 to 80.216 µmol·min^−1^·g^−1^ dry cell mass in six cycles (80.216, 67.948, 57.990, 41.451, 50.676, 42.777 µmol·min^−1^·g^−1^ dry cell mass). In the control group without IM328_sqr cells, sodium sulfide gradually increased, leading to an accumulation in content. The result as mentioned above indicated that the heterotrophic IM328 expressing heterologous *sqr* had a high and stable rate for sulfide biotreatment after recycling for at least six times.

## Discussion

In this study, hundreds of sulfide oxidation genes were mined from the metagenomes of soda-saline lakes (Fig. 1), and an engineered haloalkaliphilic bacterium overexpressing the sulfide oxidation gene *sqr* was developed for biodesulfurization (Fig. 5). Hydrogen sulfide is an acid gas, so alkaline conditions are more conducive for the absorption of hydrogen sulfide (de Rink et al. 2020). In addition, hydrogen sulfide exhibits low toxicity to microorganisms under alkaline conditions (Wu et al. 2020). We found that the optimal pH of 8.0 for the growth of alkaliphilic *H*. *salifodinae* IM328 was not the best for the sulfide oxidation, but that increased to 9.0 (Figs. 2e and 6b). This means that the elevated pH value could have enhanced the cell activities for desulfurization. This can possibly be due to the reduced toxicity of hydrogen sulfide to the cells. Therefore, alkaliphilic bacteria and their genetic resources have great application potentials in the desulfurization treatment in such a high pH environment. However, extreme alkaline conditions may not be necessary for desulfurization. Our result indicated that the higher pH of 10.0 would not further increase sulfide oxidation (Fig. 6b). Moreover, if the system is too alkaline, it will also cause environmental pollution and increase the process cost.

The haloalkaliphiles have been researched for removing hydrogen sulfide in the saline and alkaline system. For example, the autotrophic bacterium *Thioalkalivibrio versutu*s (Xu et al. 2015) and even more significant *Thioalkalivibrio sulfidophilus* have been identified as the dominant SOB in sulfide-oxidizing bioreactors (Sorokin et al. 2015). *Thiobacillus* was also employed in previous researches because organic carbon source addition is not required for desulfurization (Tang et al. 2009). As we know, the haloalkaliphilic chemoautotrophic sulfur-oxidizing bacterium is used in the commercial biodesulfurization processes named Thiopaq^®^. Our research explores the possibility of whole-cell catalysis by haloalkaliphilic heterotrophic bacteria to remove sulfide. By comparison, heterotrophs usually grow fast and maintain high biomass. This would lead to a high rate of biodesulfurization. Although organic nutrients are needed, the industrialization of biodesulfurization based on the heterotrophs depends on the total cost, including the running cost.

As previously reported, the researchers have found that the sulfide oxidation rates of eight heterotrophic bacteria ranged from 0.1 to 50 µmol·min^−1^·g^−1^ dry cell mass. Especially heterotrophic *Gluconobacter oxydans* 621 H oxidizes sulfide at a rate as high as 50 µmol·min^−1^·g^−1^ dry cell mass (Hou et al. 2018). Considering the cost of cells culture by addition of organic compounds, the whole-cell catalytic desulfurization process was performed to offset this issue. In the present study, haloalkaliphilic *H*. *salifodinae* IM328 showed the oxidation rate of sulfide ranging from 41.451 to 80.216 µmol·min^−1^·g^−1^ dry cell mass in the whole-cell catalytic desulfurization system of pH 9.0 (Fig. 7). The sulfide oxidation rate is equivalent to the highest reported value. Moreover, magnetically immobilized heterotrophs on Fe_3_O_4_ nanoparticles were also recyclable and convenient to solve the high cost of recycling heterotrophic bacterial cells (Hou et al. 2018). However, it was reported that these immobilized microbes could keep the desulfurization ability through re-incubation of 12 h in a fresh medium (Hou et al. 2018). In our opinion, further research should focus on keeping the high cell activity or adopting the robust species.

Additionally, it has been reported that Sqr and Pdo oxidize sulfide to polysulfide and further to thiosulfate in most bacteria during aerobic growth (Xia et al. 2017). Notably, Sqr (KO: K17218) was annotated, but no Pdo was found in the genome of the bacterium IM328. Thus, sulfide could be oxidized to zero-valent sulfur or polysulfide by IM328 but could not be further oxidized to thiosulfate, sulfite, or sulfate. Logically, the oxidation process will not affect the pH value of the whole-cell system. Correspondingly, we observed that the pH did not decrease even after six cycles (data not shown). This advantage would reduce the cost of the whole-cell catalyst system. Even though, the product of sulfide oxidation needs further systematic research in the future. Another problem is that the heterologous expression of *sqr* by chemoheterotrophic bacteria requires oxygen as the final electron acceptor. This leads to safety risks when operating the reactor under oxygen-rich conditions, especially when processing gas streams such as natural gas or biogas. It is necessary to control the amount of oxygen introduced in the natural gas system’s hydrogen sulfide treatment process. It is also possible to separate the absorption process from the bioreactor or pass natural gas and oxygen in turn. In addition, further metabolic modification can be considered to enable the engineered strains to use nitrate as an electron acceptor for sulfide oxidation under anaerobic conditions (Mahmood et al. 2007). This will reduce the operating cost and safety risks of the aeration process.

To enhance the desulfurization efficiency, we performed the process optimization and strain’s improvement (Fig. 6). Both strategies are effective in improving the biodesulfurization rate. A new dual-bioreactor including an anaerobic bioreactor line-up in the biological gas desulfurization process under saline and alkaline conditions improves the selectivity for the sulfur formation and the removal efficiency of HS^−^. Due to the anaerobic reactor, the SOB could remove HS^−^ (de Rink et al. 2020). In terms of the development of bacteria for desulfurization by metabolic engineering, expression of *Vitreoscilla* globin gene (*vgb*) and knockout of sulfate producing key gene *hdrB* improved desulfurizing activity using haloalkaliphilic *T*. *versutus* (Mu et al. 2017; Sharshar et al. 2020). Fed-batch culture coupled with design, build, test, and validate approach successfully led to the first tight inducible system construction in *T*. *versutus* for improving biodesulfurization processes (Sharshar et al. 2020). In our research, enhancing the metabolic activity by overexpression of the *sqr* gene could increase the sulfide oxidation rate by 34 %. Due to the lack of genetic tools like promoters, we could not optimize it further.

A point of concern is that there are plasmid and antibiotic resistant gene in the engineered strain, and the leakage of microorganism may threaten biosafety (Zhang et al. 2019). Alternatively, the *sqr* gene could be expressed through integrating into the chromosome. This manipulation would not only make the engineered strain more stable but also improve biosafety. During the experiment, we used mass spectrometry to characterize the expression, because its level was too low and a large quantity of different bands were observed in the SDS-PAGE (Fig. 5). The mass spectrometry is sensitive to detect the protein. Some of the multiple bands may be the contaminations from the other band (Fig. 5, Additional file 1: Table S3). The expression of the *sqr* gene was also verified by further measuring the increase in enzyme activity (Fig. 6a). In summary, we developed a whole-cell catalyst of an engineered haloalkaliphilic bacterium and exhibited the possibility of commercialization for biodesulfurization. Meanwhile, more researches on strain improvement and downstream processes are proposed further.

## Supplementary Information

**Additional file 1: Table S1.** Reference sequences of different types of Sqr and FCSD used for evolutionary analysis. **Table S2.** Sequence data of 484 Sqrs and FCSDs used in the evolutionary tree. **Table S3.** Results of mass spectrometry.

**Additional file 2: Figure S1. **Maximum Likelihood phylogenomic tree based on concatenated amino acid sequences of 120 conserved proteins. **Figure S2.** The standard curve of sulfide concentration.

## Data Availability

The GenBank accession number of *Halomonas salifodinae* IM328 genome sequence is NZ_JADOTW000000000, and that of Sqr sequence from *Spiribacter* sp. IM2438 is WP_154297083.1.
