# Supplementary material for: Development of whole-cell catalyst system for sulfide biotreatment based on the engineered haloalkaliphilic bacterium
Source: AMB Express. 2021 Oct 24;11:142. doi: 10.1186/s13568-021-01302-9 (PMC8542531; doi:10.1186/s13568-021-01302-9)
Supplement: Supplementary file 2 — Additional file 2: Figure S1. Maximum Likelihood phylogenomic tree based on concatenated amino acid sequences of 120 conserved proteins. Figure S2. The standard curve of sulfide concentration. [file 13568_2021_1302_MOESM2_ESM.pdf]

Supplementary materials

Development of whole-cell catalyst system for sulfide biotreatment based on the engineered haloalkaliphilic bacterium

Manqi Zhang[1,2], Qiong Xue[1,2], Shengjie Zhang[1,2], Heng Zhou[1], Tong Xu[1], Jian Zhou[1], Yanning Zheng[1], Ming Li[1], Sumit Kumar[3], Dahe Zhao[1*], Hua Xiang[1,2*]

Author affiliations:

[1] State Key Laboratory of Microbial Resources, Institute of Microbiology, Chinese Academy of Sciences, 100101, Beijing, China

[2] University of Chinese Academy of Sciences, 100049, Beijing, China

[3] Enzyme and Microbial Biochemistry Lab, Department of Chemistry, Indian Institute of Technology, Delhi, India

Corresponding authors:

Hua Xiang, xiangh@im.ac.cn; Dahe Zhao, zhaodh@im.ac.cn

Supplementary figures

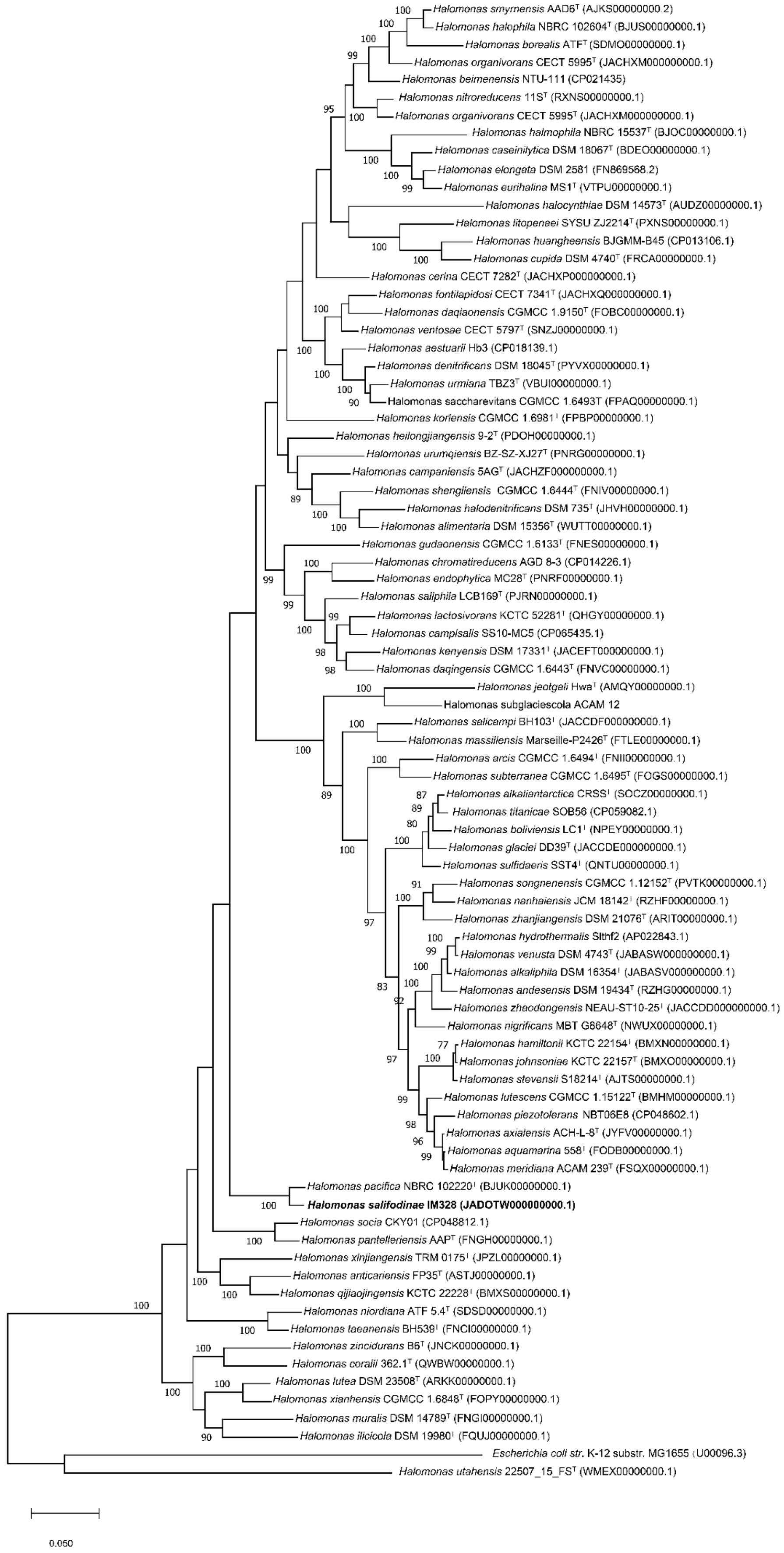

Fig. S1 Maximum Likelihood phylogenetic tree based on concatenated amino acid sequences of 120 conserved proteins. Bootstrap values (%) were based on 1000 replicates and shown with more than 70% bootstrap support. Bar, 0.050 substitutions per amino acid position.

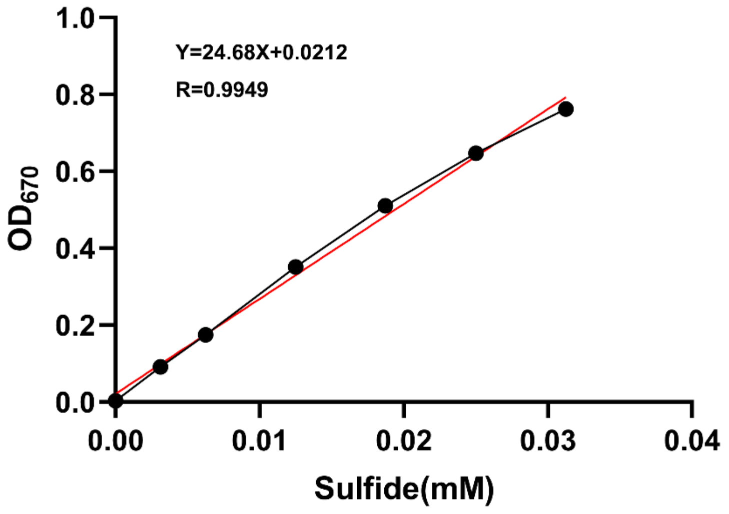

Fig. S2 The standard curve of sulfide concentration. In seven 15 mL capped centrifuge tubes, different ratios of 10 mg/L sulfide standard solution (0, 0.1 mL, 0.2 mL, 0.4 mL, 0.6 mL, 0.8 mL, 1.0 mL, respectively) and distilled water were added in turn to make the total volume to 10 mL. After the mixtures were thoroughly mixed, theirs absorbances were measured spectrophotometically at 670 nm using a diamine reagent. The equation was calculated by linear regression.
